# Evaluating and Screening of Agro-Physiological Indices for Salinity Stress Tolerance in Wheat at the Seedling Stage

**DOI:** 10.3389/fpls.2021.646175

**Published:** 2021-03-31

**Authors:** Rongrong Tao, Jinfeng Ding, Chunyan Li, Xinkai Zhu, Wenshan Guo, Min Zhu

**Affiliations:** ^1^Jiangsu Key Laboratory of Crop Genetics and Physiology, Agricultural College of Yangzhou University, Yangzhou, China; ^2^Jiangsu Key Laboratory of Crop Cultivation and Physiology, Agricultural College of Yangzhou University, Yangzhou, China; ^3^Co-Innovation Centre for Modern Production Technology of Grain Crops, Yangzhou University, Yangzhou, China

**Keywords:** wheat, salt tolerance, leaf K^+^/Na^+^ ratio, Na^+^ exclusion, stomatal density, osmotic adjustment

## Abstract

Soil salinity is a worldwide issue that affects wheat production. A comprehensive understanding of salt-tolerance mechanisms and the selection of reliable screening indices are crucial for breeding salt-tolerant wheat cultivars. In this study, 30 wheat genotypes (obtained from a rapid selection of 96 original varieties) were chosen to investigate the existing screening methods and clarify the salinity tolerance mechanisms in wheat. Ten-day-old seedlings were treated with 150 mM NaCl. Eighteen agronomic and physiological parameters were measured. The results indicated that the effects of salinity on the agronomic and physiological traits were significant. Salinity stress significantly decreased K^+^ content and K^+^/Na^+^ ratio in the whole plant, while the leaf K^+^/Na^+^ ratio was the strongest determinant of salinity tolerance and had a significantly positive correlation with salt tolerance. In contrast, salinity stress significantly increased Na^+^ concentration and relative gene expression (*TaHKT1;5*, *TaSOS1*, and *TaAKT1-like*). The Na^+^ transporter gene (*TaHKT1;5*) showed a significantly greater increase in expression than the K^+^ transporter gene (*TaAKT1-like*). We concluded that Na^+^ exclusion rather than K^+^ retention contributed to an optimal leaf K^+^/Na^+^ ratio. Furthermore, the present exploration revealed that, under salt stress, tolerant accessions had higher shoot water content, shoot dry weight and lower stomatal density, leaf sap osmolality, and a significantly negative correlation was observed between salt tolerance and stomatal density. This indicated that changes in stomata density may represent a fundamental mechanism by which a plant may optimize water productivity and maintain growth under saline conditions. Taken together, the leaf K^+^/Na^+^ ratio and stomatal density can be used as reliable screening indices for salt tolerance in wheat at the seedling stage.

## Introduction

Soil salinization is one of the main abiotic stress factors affecting crop yields worldwide; approximately 6% of the world’s total land area is threatened by salinity, including 20% of arable land and 33% of irrigated land (Shrivastava and Kumar, 2015; Kuang et al., 2019; Safdar et al., 2019). Furthermore, land salinization is increasing, with 10 million ha of agricultural land destroyed annually by salt accumulation due to human activity and other factors related to climate change (Smajgl et al., 2015; Isayenkov, 2019). Salinity stress significantly decreases plant growth and productivity, which can substantially reduce yield production (Munns et al., 2019). Wheat (*Triticum aestivum* L.) is one of the most important crop plants worldwide and feeds a large number of people. However, wheat is only moderately tolerant to salinity; the loss in its grain yield exceeds 60% under saline conditions (Khan et al., 2017). One of the most effective and feasible ways to minimize the detrimental effects of salinity on crop production is to enhance the salinity-tolerant ability (Sergey, 2013; Luo et al., 2019).

Salinity tolerance is a complex physiological trait, composed of many sub-components; the classical view is that salinity affects plant performance via osmotic stress and specific ion toxicity (Munns and Tester, 2008). Elevated NaCl levels in the soil solution affect the ability of plants to take up water, which can be observed immediately after salt application, and this is believed to dominate for a few weeks (Munns, 2002). Generally, plants overcome lower osmotic potential in the rhizosphere by reducing water loss. It has been argued that stomatal transpiration accounts for about 95% of plant water loss (Hedrich and Shabala, 2018). Salinity-induced oxidative damage occurs because of the presence of excessive reactive oxygen species (ROS), but a low concentration of ROS has beneficial impacts at the cellular level, e.g., H_2_O_2_ functions as a signaling molecule, controlling the stomatal aperture with abscisic acid (ABA). Besides, a high concentration of ABA and H_2_O_2_ under saline conditions can activate potassium and anion efflux to guard the cell, resulting in transient stomatal closure (Hedrich and Shabala, 2018). This will help reduce transpiration to help plants overcome osmotic stress caused by salt stress. It has been reported that lower stomatal density is an important physiological trait in salinity-tolerant quinoa (Shabala et al., 2012, 2013), and cultivated barley tends to employ a stress-escaping strategy by reducing stomatal density to conserve water when grown under saline conditions (Kiani-Pouya et al., 2020). However, salinity-induced stomatal closure would limit CO_2_ influx, inhibiting leaf photosynthetic capacity and ultimately yield (Lawlor and Wilmer, 2009). Is the stress-escaping strategy adopted by wheat to reduce water loss under salt stress? Is there a corresponding compensation mechanism to reduce production loss? These questions need further exploration.

The primary challenge for plants under salt stress is ionic toxicity, which is caused by the excessive accumulations of sodium in the cytoplasm (Su et al., 2020). Na^+^ exclusion from the shoot is considered important for a plant to counteract the detrimental effects of increased salinity, and a major portion of Na^+^ exclusion (>98%) in wheat is accomplished by restricting net Na^+^ uptake at the soil-root interface and net xylem loading in roots (Tester and Davenport, 2003). Furthermore, the K^+^/Na^+^ ratio is considered the key feature conferring salinity stress tolerance in plants, which is often considered a potential screening tool for plant breeders (Munns and Tester, 2008; Genc et al., 2016; Oyiga et al., 2018). Na^+^ and K^+^ transporters are vital for maintaining the homeostasis of Na^+^ and K^+^ in cells and plants under salt stress (Azhar et al., 2017), i.e., SOS (salt overly sensitive), HKT (high-affinity K^+^ transporter), and AKT1, an inward-rectifying K^+^ channel. As one of the large superfamilies of ionic transporters, high-affinity potassium transporters (HKTs) play physiological roles in salinity tolerance through the removal of Na^+^ from the xylem under salt stress (Su et al., 2015). HKT genes can be classified into two main classes based on their transport selectivity. Class 1 reduces the transport of Na^+^ to aboveground tissues, while Class 2 plays a crucial role in Na^+^ and K^+^ transport (Munns and Tester, 2008). In addition to HKT genes, the SOS pathway is commonly viewed as a key pathway involved in the regulation of Na^+^ homeostasis in plants through the activity of the genes SOS1, SOS2, and SOS3 (Katschnig et al., 2015). Of these, SOS1 is a Na^+^/H^+^ antiporter and stimulates efflux Na^+^ in exchange for H^+^ (Gong et al., 2004). AKT1 was first cloned from *Arabidopsis thaliana* and characterized as an inward-rectifying K^+^ channel with high selectivity for K^+^ over Na^+^, and *TaAKT1* is orthologous to *AtAKT1* (Buschmann et al., 2000). AKT1 channels are expressed predominantly, but not exclusively, in the roots. It has been argued that AKT1 overexpression improves osmotic and stress tolerance by increasing tissue levels of K^+^ (Ahmad et al., 2016). With advances in molecular biology and biotechnology tools, the roles of *TaHKT1;5* and *TaSOS1* in Na^+^ exclusion and its mechanisms have been exhaustively characterized in previous research (Zhu et al., 2016a; Wu et al., 2018; Ahmadi et al., 2020). However, there are few studies on *TaAKT1*, and its mechanism in maintaining K^+^ homeostasis under salt stress needs further research.

Less than 25% of salt-regulated genes are salt stress-specific imparting the most basic characteristics of key physiological characteristics, and still the first choice for plant salt-tolerant breeding (Shabala et al., 2013). Identification of suitable screening agro-physiological parameters that have potential as screening criteria for discriminating wheat genotypes for salt tolerance is a prerequisite in the production of resistant wheat genotypes (El-Hendawy et al., 2017). Field yield was the most commonly used and simplest screening technique for salt-tolerant varieties in previous studies. However, field-based trials may not be easy and can be problematic due to the complexity of environmental conditions. Hence, attention has focused on the salinity stress of wheat plants at the seedling stage as opposed to the entire life cycle of plants under controlled environmental conditions, with biomass production used as an assessment of salinity tolerance (Munns and James, 2003). Based on relative biomass, a new stress tolerance index (STI) was defined, which had a positive and significant correlation with the potential yield under non-stressed or stress conditions and determined the most tolerant wheat genotypes (Talei et al., 2013; Singh et al., 2015). The maintenance of photosynthetic activity under saline conditions can be used to identify salt-tolerant genotypes (Munns and Gilliham, 2015). In this context, different photosynthetic parameters such as the chlorophyll content and maximum quantum PSII photochemical efficiency (F_v_/F_m_) have been confirmed as the key physiological traits of salt tolerance. For instance, a positive correlation between the chlorophyll content and the overall plant salinity tolerance has been reported in both barley and wheat (Wu et al., 2015). Further, early detection of chlorophyll fluorescence was used as a means to prevent the loss of plant biomass under high salinity conditions (El-Hendawy et al., 2019).

Despite intensive efforts, little success has been achieved in breeding cultivars tolerant to saline conditions. The lack of precise indices of physiological and agronomic traits related to salinity stress and the low genetic variability of currently available wheat germplasm are among the main reasons for the limited success in breeding salt-tolerant wheat varieties (Ismail and Horie, 2016; Miransari and Smith, 2019). Besides, multi-parameter analysis is accompanied by complex statistical methods that make the operation inconvenient and inefficient (Oyiga et al., 2016). Moreover, the evaluation of physiological traits is also dependent on the researcher’s personality. The objective of this study was to identify the important agronomic and physiological characteristics associated with salt tolerance to develop a rapid and feasible assay for salt tolerance in wheat and to reveal the relationships between all of the tested parameters as well as to identify the parameters that can be employed as reliable screening indices for the selection and improvement of salt-tolerant cultivars.

## Materials and Methods

### Plant Materials

Thirty wheat cultivars (selected from varieties grown on salt-stressed soils in China and northern Sudan; a short description is given in Supplementary Table 1) were obtained from the Wheat Research Centre of Yangzhou University and were used in this study. The full list of cultivars is given in Table 1.

**TABLE 1:** The list of wheat varieties used in this study.

**No.**	**Genotype**	**Origin**	**No.**	**Genotype**	**Origin**
1	Argine	Sudan	16	Ningmai 23	China (Jiangsu)
2	Buahin	Sudan	17	Ningmai 24	China (Jiangsu)
3	Elnilein	Sudan	18	Xianmai 8	China (Henan)
4	Annong 1124	China (Anhui)	19	Xiangmai 25	China (Hubei)
5	Emai 352	China (Hubei)	20	Xumai 33	China (Jiangsu)
6	Emai 195	China (Hubei)	21	Yannong 19	China (Shangdong)
7	Emai 251	China (Hubei)	22	Yannong 999	China (Shangdong)
8	Emai 580	China (Hubei)	23	YFM 4	China (Jiangsu)
9	Huamai 6	China (Jiangsu)	24	Yangmai 20	China (Jiangsu)
10	Huamai 7	China (Jiangsu)	25	Yangmai 21	China (Jiangsu)
11	Huaimai 29	China (Jiangsu)	26	Yangmai 23	China (Jiangsu)
12	Lemai G1302	China (Anhui)	27	Yangmai 25	China (Jiangsu)
13	Lianmai 7	China (Jiangsu)	28	Zhenmai 11	China (Jiangsu)
14	Ningmai 21	China (Jiangsu)	29	Zhengmai 119	China (Henan)
15	Ningmai 22	China (Jiangsu)	30	Zhengmai 9023	China (Henan)

### Experimental Design

The study was conducted twice during 2018–2019 in a glasshouse at the Agricultural College of Yangzhou University (32°39’E, 119°42’N) in China. The day/night temperatures were about 24/16 ± 2°C. Seven seeds of each genotype were sown in a 4 L pot filled with a standard potting mixture (refer to Chen et al., 2007). Ten days after sowing, a salinity treatment was started by watering plants with a 150 mM NaCl solution. The treatment was applied several times until the solution running out of the pot reached a salt level of 150 mM. The control pots were irrigated with tap water and were grown in the same glasshouse, with three replicates. Four weeks after the treatment, treated and control plants were assessed for various physiological traits described below. Abbreviations of the traits in the study are given in Table 2.

**TABLE 2:** The list of traits tested in salt tolerance evaluation.

**Abbreviations**	**Description**
STI	Salt tolerance index based on the total dry weight
Chl	Chlorophyll content (SPAD values)
F_v_/F_m_	Chlorophyll fluorescence
SD	Stomatal density (cells/mm^2^)
OSM	Leaf sap osmolality (mmol/kg)
PH	Plant height (cm)
RL	Root length (cm)
SDW	Shoot dry weight (g)
RDW	Root dry weight (g)
SFW	Shoot fresh weight (g)
RFW	Root fresh weight (g)
SWC	Shoot water content (%)
RWC	Root water content (%)
Leaf K	Leaf K^+^ content (mmol/L)
Leaf Na	Leaf Na^+^ content (mmol/L)
Leaf K_Na_R	Leaf K^+^/Na^+^ Ratio
Root K	Root K^+^ content (mmol/g)
Root Na	Root Na^+^ content (mmol/g)
Root K_Na_R	Root K^+^/Na^+^ Ratio

### Sampling and Measurements

#### Chlorophyll Content

The first fully expanded leaf was measured using a SPAD-502 (Konica Minolta, Osaka, Japan). Ten points were measured evenly on each leaf. The 10 readings per leaf were averaged. Measurements were conducted on five plants of each cultivar exposed to salinity stress and the control. The highest and lowest values were discarded during analyses, and the remaining data were averaged.

#### Chlorophyll Fluorescence Measurements

The maximum photochemical efficiency of PSII was estimated by measuring the chlorophyll fluorescence F_v_/F_m_ ratio using a pulse-amplitude modulation portable fluorometer Mini-PAM-II (Walz, Effeltrich, Germany) (as described in Smethurst et al., 2008). Five replicates per cultivar for both treated and control plants were assessed.

#### Stomatal Density

The abaxial leaf surface was coated with clear nail varnish. The dried layer of the nail varnish was then peeled off using tweezers and placed on a glass slide. These imprints were later examined under an optical microscope with a 40× objective lens, and stomatal density (number of cells per surface area) was determined. The sample size for each treatment and genotype was 45 (three epidermal samples per leaf × three fields of view per sample × five biological replicates). The highest and the lowest values were discarded during analyses, and the remaining data were averaged.

#### Leaf Sap Osmolality and Na^+^ and K^+^ Contents

The youngest fully expanded leaf was harvested and rapidly frozen in an Eppendorf tube using liquid nitrogen. To measure the ion content and osmolality, the leaf was defrosted and the sap extracted by hand-squeezing the leaf samples. The sap was centrifuged at 8,000 rpm for 10 min using a high-speed centrifuge (Centrifuge 5415D, Eppendorf, Germany) to remove debris. An amount of 20 μl of the collected supernatant was used to measure sap osmolality using a dew-point osmometer (Vapro 5600, WESCO, United States). An additional 50 μl of the collected supernatant was made up to 5 mL with distilled water to determine the K^+^ and Na^+^ concentrations (in mM) using a flame photometer (Corning 410C, Essex, United Kingdom). Five replicates per cultivar for both salt-treated and control plants were assessed.

#### Root Length, Plant Height, and Biomass of the Shoot and Root

The whole plant was divided into the shoot and root after harvesting. The distances from the crown to the leaf tip and root tip were measured as the plant height and root length, respectively. Root length and plant height were measured using a ruler. Fresh weight (FW) was determined using an electronic balance. Fresh samples were then washed with deionized water and dried at 80°C to a constant weight (DW), and the water content (WC) was calculated with the following formula: WC = (FW – DW)/ FW × 100.

#### Root Na^+^ and K^+^ Contents

The dried root samples were digested with H_2_NO_3_–H_2_O_2_ in a microwave digestion system (MARS5, CEM, United States). The digestion solution was adjusted to 50 ml with distilled water to determine the K^+^ and Na^+^ concentrations (in mM) using a flame photometer (Corning 410C).

#### RNA Extraction and RT-qPCR Experiment

Salt-tolerant wheat (Huaimai 29) and salt-sensitive wheat (Argine) were selected based on the results of this research and were used in this experiment. Seeds were surface-sterilized with 5% NaClO for 10 min, rinsed thoroughly under running tap water, and then grown hydroponically with distilled water in a dark growth cabinet at 23 ± 2°C.

Six-day-old hydroponically grown seedlings, treated with 150 mM NaCl for 24 h, were harvested and snap-frozen in liquid nitrogen, and the total RNA of the shoot was extracted using an RNA simple Total RNA kit (Tiangen Biotech, Beijing, China) according to the manufacturer’s instructions. First-strand cDNA was synthesized using the HiScript II Q RT SuperMix for qPCR (+gDNA wiper) (Vazyme Biotech, Nanjing, China). Quantitative real-time PCR was performed on the cDNA for the transporters *TaHKT1;5* (GenBank: U16709.1), *TaSOS1* (GenBank: U16709.1), and *TaAKT1-like* (GenBank: AF207745.1) using the ChamQ Universal SYBR qPCR Master Mix (Vazyme Biotech, Nanjing, China) in a CFX Connect real-time PCR system (BIO-RAD, United States). Each analysis had three biological repeats with three technical replicates. *TaActin* (GenBank: AB181991.1), which exhibited a constant expression level in all of the samples, was used as an internal control to normalize the expression of the target genes. The 2^–ΔΔCT^ method was used to analyze the relative expression levels of the studied genes (as described in Livak and Schmittgen, 2001). Primer sequences are shown in Supplementary Table 2.

### Statistical Analysis

The experiment was performed twice, and there were no significant differences in all variables between the two experiments; therefore, the average of each variable of the two experiments was used for data analysis. The salt tolerance index (STI) of each variety was determined as a ratio of the total dry weight under salt treatment relative to the total dry weight of the control.

Microsoft Excel 2016 was adopted for data processing and figure drawing. Statistical analyses were performed with SPSS statistical 26. All of the data were subjected to one-way analysis of variance (ANOVA) followed by means comparison using Duncan’s multiple range test (*p* < 0.05). In the correlation analysis, the non-parametric Spearman test was used for the correlation of 18 traits, and the *Pearson* correlation coefficient was used to analyze the correlation between STI and other traits. The stepwise method was used for multiple linear regression; STI was used as a dependent variable, and all studied characteristics were used as independent variables. Three different scores of all 18 traits were used for regression analysis. These included values under control conditions (C), values under saline stress (S), and relative values under saline stress (% control) (R).

## Results

### Tolerance of all Varieties Under Salinity Stress

**FIGURE 1:**
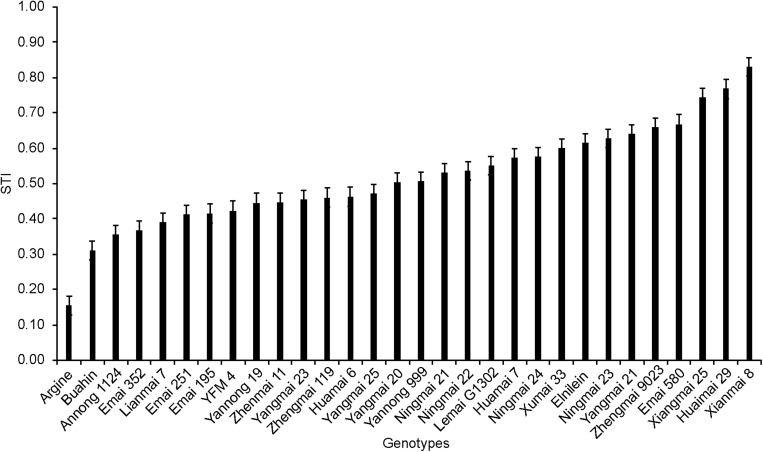
The salt tolerance index (the ratio of the dry plant weight subjected to 150 mM NaCl treatment relative to the dry plant weight of the control) of 30 wheat varieties.

The STI of 30 wheat varieties subjected to 150 mM NaCl is shown in Figure 1. Varieties showed significant differences in salt tolerance, with the STI varying from 0.15 to 0.83. Xianmai 8 showed better tolerance than the other varieties.

### Comparative Analysis of Traits of Salt-Treated and Control Seedlings

**FIGURE 2:**
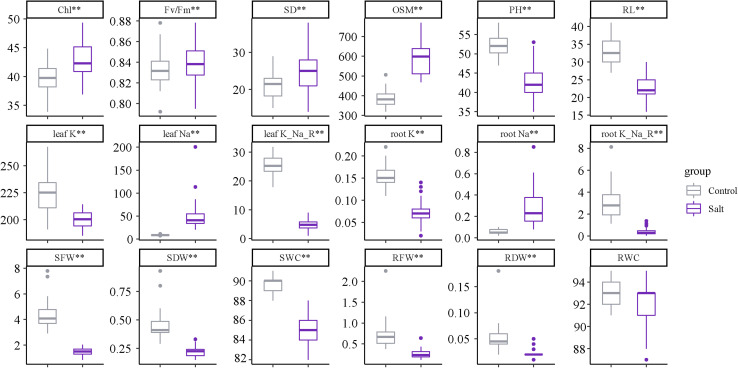
Trait performance under normal and salt treatments. Asterisks (**) indicate that the trait mean was significantly different (*p*-value < 0.01) between the salt and control conditions.

Four weeks of salinity stress resulted in significant (significant at *p* < 0.01) changes in the physiological and agronomic characteristics (Figure 2). Different from previous research results, the leaf sap osmolality, chlorophyll content, stomatal density, and F_v_/F_m_ showed significant increases as well as the Na^+^ content under the treatment. Salinity stress also affected plant growth and biomass production, resulting in a significant reduction.

**FIGURE 3:**
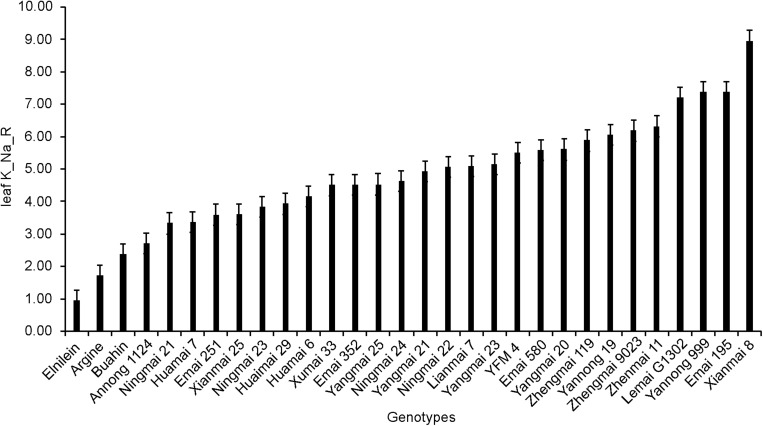
Leaf K^+^/Na^+^ ratio of 30 wheat varieties under 150 mM NaCl stress.

Similar to the STI, the average performance of different physiological traits also varied significantly among the cultivars. Varieties showed extremely significant differences in ion loading, with the leaf K^+^/Na^+^ ratio varying from 0.96 to 8.95 (Figure 3).

### Correlation Between STI and Different Agro-Physiological Traits Under Salinity Stress

**FIGURE 4:**
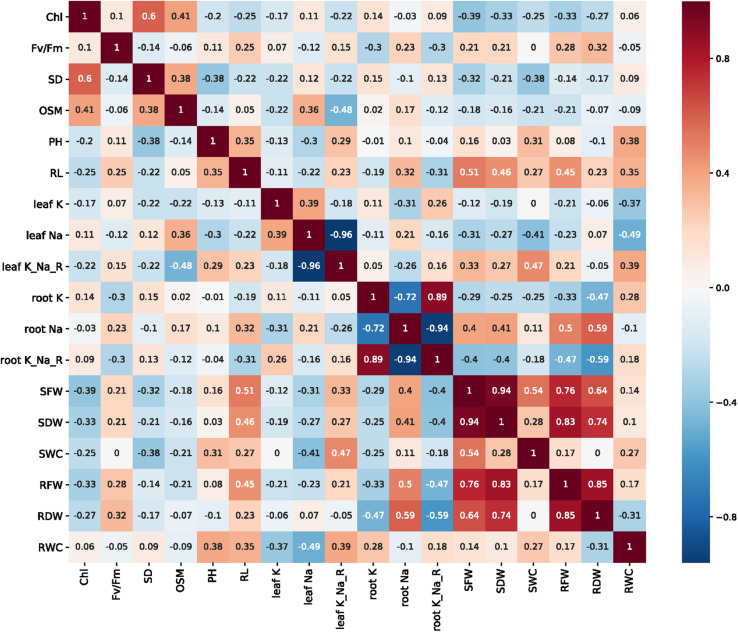
Correlation map of the 18 traits based on Spearman correlation analysis (positive, red; negative, blue. Non-significant (*p* ≥ 0.05) values are indicated by blank blocks).

Most agro-physiological traits were found to correlate with each other, and the correlation between different traits is shown in Figure 4. Biomass traits (wet and dry weight, water content) had significant correlations with the Na^+^ content, K^+^ content, and K^+^/Na^+^ ratio, among which the water content was positively correlated with the leaf K^+^/Na^+^ ratio (*p* < 0.01). The correlation between the leaf sap osmolality and biomass traits was not significant; however, the leaf sap osmolality was positively associated with the chlorophyll content and stomatal density, while a significant negative correlation was observed between osmolality and the leaf K^+^/Na^+^ ratio (at *p* < 0.01).

Table 3 shows the correlations between various agro-physiological traits and the STI under 150 mM NaCl for all 30 wheat genotypes. The leaf sap osmolality, shoot water content, leaf Na^+^ content, and leaf K^+^/Na^+^ ratio all showed significant correlations with the STI. Among the traits assessed, the leaf sap osmolality had a negative relationship with the STI. In further analysis, the leaf sap osmolality under salt/control conditions still had a significant correlation with STI, while the leaf K^+^/Na^+^ ratio was the strongest determinant of salinity tolerance. Based on a comparison of the results of the salt-treated values, under salt/control conditions, the shoot dry weight and fresh weight showed significant correlations with the STI (*p* < 0.05), while the chlorophyll content, root length, and stomatal density showed no significant correlations (*p* > 0.05).

**TABLE 3:** The correlations between the salt tolerance index (STI) and different agro-physiological traits (*p-value < 0.05, **p-value < 0.01).

	**Salt**	**Control**	**Salt/Control**
Chl	0.529**	0.550**	0.057
F_v_/F_m_	0.155	0.073	0.175
SD	−0.346*	−0.489**	−0.022
OSM	−0.681**	−0.450**	−0.384**
PH	0.028	−0.156	0.153
RL	0.251*	−0.268	0.318
SDW	0.001	−0.562**	0.356*
RDW	−0.146	−0.384*	0.025
SFW	0.294	−0.499**	0.427*
RFW	−0.012	−0.392*	0.227
SWC	0.606**	0.443**	0.467**
RWC	0.271	−0.047	0.269
leaf K	−0.254	0.044	−0.22
leaf Na	−0.513**	−0.051	−0.499**
leaf K_Na_R	0.466**	0.063	0.504**
Root K	−0.067	0.302	−0.246
Root Na	−0.037	0.004	−0.046
Root K_Na_R	0.04	0.281	−0.113

### Agro-Physiological Traits That Had a Significant Contribution to Salt Tolerance

**FIGURE 5:**
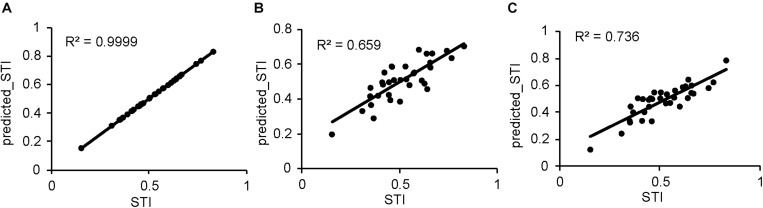
Correlations between actual STI and the STI predicted from the agro-physiological traits with significant contributions to salinity tolerance (Table 4). **(A)** Trait ratios. **(B)** Traits under salt stress. **(C)** Traits under the control condition.

Many agro-physiological traits showed a significant correlation with the STI (Table 3). To find the most important traits that made major contributions to salt tolerance, a linear regression analysis was conducted using three different scores (values under control and the salt-treated and relative values) of all of the agro-physiological traits. As shown in Table 4, when the analysis was based on the relative values in the saline treatment (% control), eight agro-physiological traits showed significant contributions to the tolerance, and significant correlations were found between the actual STI and the STI predicted using different agro-physiological traits (*R*^2^ = 0.99, Figure 5A). Shoot dry weight was the major contributor to salt tolerance (Table 4). Further analysis was conducted on the values under the saline treatment and control conditions. Under salinity stress, the stomatal density, plant height, shoot dry weight, and shoot water content had significant contributions to the STI, with the shoot water content being the most important trait. Together, these four traits determined more than 66% of the relative STI (Figure 5B). In the analysis of the control values, six agro-physiological traits showed significant contributions to tolerance and determined 74% of the STI variation (Figure 5C).

**TABLE 4:** Coefficients of the agro-physiological traits with a significant contribution to STI.

	**Salt**	**Control**	**Salt/Control**
	**(*R*^2^ = 0.66)**	**(*R*^2^ = 0.74)**	**(*R*^2^ = 0.99)**
Intercept (b)	−2.112	0.411	0.105
Chl	–	−0.016	−0.022
SD	0.01	0.019	–
PH	−0.009	–	–
leaf K	–	0.002	–
Root K	–	2.019	–
SDW	2.56	−1.32	0.914
RDW	–	–	0.073
SFW	–	–	−0.025
RFW	–	0.184	0.030
SWC	3.155	–	–
RWC	–	–	−0.084
Root K	–	–	0.009
Root K_Na_R	–	–	−0.013

**FIGURE 6:**
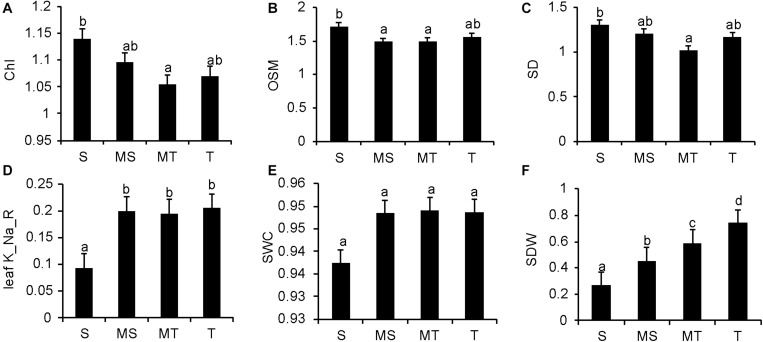
Mean performance of the significant traits (selected in the regression analysis and correlation analysis; Tables 3, 4) in the four clusters. Cluster 1, sensitive (S; STI < 0.35); Cluster 2, moderately sensitive (MS; STI 0.35-0.50); Cluster 3, moderately tolerant (MT; STI 0.50-0.65); Cluster 4, tolerant (T; STI > 0.65). **(A–F)** show the mean performances of chlorophyll content, leaf sap osmolality, stomatal density, leaf K^+^/Na^+^ ratio, shoot water content and shoot dry weight in the four clusters, respectively. Data are presented as the mean ± SE (*n* = 5). Different lowercase letters indicate a significant difference (*P* < 0.05).

In additional statistical analysis, all cultivars were divided into four groups: sensitive (S – STI < 0.35); moderately sensitive (MS – STI ranging from 0.35 to 0.50); moderately tolerant (MT – STI ranging from 0.50 to 0.65), and tolerant (T – STI ranging from > 0.65) (Table 5). The average values of the selected agro-physiological traits that had significant correlations with STI and showed significant contributions to STI in the above regression analysis and correlation analysis are shown in Figure 6. Compared with salt-sensitive genotypes, salt-tolerant genotypes had a higher shoot dry weight, shoot water content and leaf K^+^/Na^+^ ratio, and the difference in the leaf K^+^/Na^+^ ratio and shoot dry weight between the salt-sensitive and salt-tolerant genotypes was significant. In salt-sensitive genotypes, the leaf sap osmolality, chlorophyll content, and stomatal density were slightly higher than the salt-tolerant genotypes.

**TABLE 5:** Genotype ranking according to the STI of 30 wheat varieties.

	**Genotype**	**STI**		**Genotype**	**STI**
S	Argine	0.15	MT	Yannong 999	0.51
	Buahin	0.31		Ningmai 21	0.53
	Annong 1124	0.35		Ningmai 22	0.54
				Lemai G1302	0.55
MS	Emai 352	0.37		Huamai 7	0.57
	Lianmai 7	0.39		Ningmai 24	0.58
	Emai 251	0.41		Xumai 33	0.60
	Emai 195	0.42		Elnilein	0.61
	YFM 4	0.42		Ningmai 23	0.63
	Yannong 19	0.45		Yangmai 21	0.64
	Zhenmai 11	0.45			
	Yangmai 23	0.45	T	Zhengmai 9023	0.66
	Zhengmai 119	0.46		Emai 580	0.67
	Huamai 6	0.46		Xiangmai 25	0.74
	Yangmai 25	0.47		Huaimai 29	0.77
	Yangmai 20	0.50		Xianmai 8	0.83

### Na^+^ and K^+^ Transporter Gene Expression Under Salinity Stress

**FIGURE 7:**
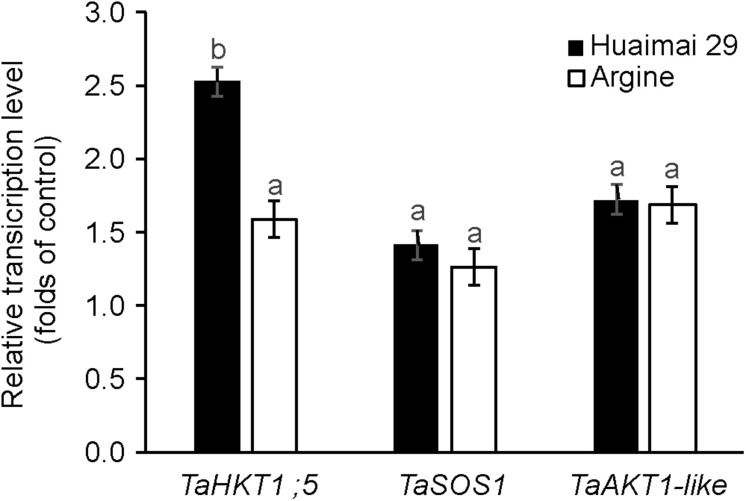
The relative gene expression in shoots of salt-tolerant wheat (cv. Huaimai 29) and salt-sensitive wheat (cv. Argine) after exposure to salt stress (150 mM NaCl, 24 h). Mean ± SE (*n* = 3 biological replicates). Different lowercase letters indicate a significant difference (*P* < 0.05).

In a separate set of experiments, a salt-tolerant variety Huaimai 29 and a salt-sensitive variety Argine were used to explore the effect of salt stress on ion transporter gene expression (for detailed growth phenotypes, see Supplementary Figure 1). As shown in Figure 7, the transcript level of all tested genes was up-regulated (compared with the control) in salt-treated (150 mM for 24 h) Huaimai 29 shoots (2. 5-, 1. 4-, and 1.7-fold for *TaHKT1;5*, *TaSOS1*, and *TaAKT1-like*, respectively) and Argine shoots (1. 6-, 1. 3-, and 1.7-fold for *TaHKT1;5*, *TaSOS1*, and *TaAKT1-like*, respectively). The ANOVA results revealed that salinity stress affected the expression profiles of the three genes in the different varieties; *TaHKT1;5* showed a significant difference between the genotypes (significant at *P <* 0.05), while the other two genes showed no significant difference between the genotypes.

## Discussion

### Agro-Physiological Responses of Wheat to Saline Conditions

Under saline conditions, crop performance is severely affected. In saline soil, Na^+^ initially enters the root cells and is then transported to shoots via the transpiration stream in the xylem. Excess Na^+^ ions may cause a range of osmotic and metabolic issues in terms of substantial changes in various agronomic and physiological traits at different organizational levels, which are induced by osmotic stress and specific ion toxicities (Miransari and Smith, 2019). Wheat (*Triticum aestivum* L.), a moderately salt-tolerant crop, shows great variability among different genotypes in response to salt (Supplementary Figure 2). In this study, Na^+^ accumulation in both roots and shoots was significantly increased under salt treatment along with the K^+^ content but a decrease in the K^+^/Na^+^ ratio (Figure 2), indicating that damage had occurred due to osmotic stress and ion toxicity. Regardless of which stress was dominant, the significantly reduced leaf osmotic pressure showed that the salt stress caused an imbalance in the water potential, resulting in a significant reduction in plant growth and biomass accumulation (Figure 2). In this context, there was a significant decrease in the shoot water content, whereas the root water content was not significantly reduced (Figure 2), consistent with the results from previous studies (El-Hendawy et al., 2005a,b; Feng et al., 2020). The root is the initial organ that senses the saline signal when the salt content in the soil dramatically increases. The effects of salinity on the shoots are more severe than those on the roots. This discrepancy occurs because roots maintain fairly constant levels of NaCl over time by exporting NaCl to the soil or the shoots (Tester and Davenport, 2003). Another interesting observation in this study was the phenomenon of salinity-induced increases in the stomatal density, chlorophyll content, and F_v_/F_m_ (Figure 2); however, the chlorophyll content and fluorescence decreased under saline conditions (Zhu et al., 2014; Yousfi et al., 2016; El-Hendawy et al., 2017). A possible explanation is a change in the cell anatomy: a sudden increase in soil salinity causes leaf cell dimensions to change, with a greater reduction in area than in depth, which makes leaves smaller and thicker, resulting in a higher number of stomata and chloroplasts per unit of leaf area (Munns and Tester, 2008).

### Salt-Tolerant Genotypes Could Implement Effective Osmotic Adjustment by Down-Regulating Stomatal Quantity to Maintain Growth Under Salinity Stress

Wheat genotypes display significant variability in agronomic and physiological responses when subjected to salinity, and salt-tolerant genotypes generally have better performance (Moez et al., 2016). In this study, all of the agro-physiological traits were correlated with the STI (Table 3). Only those agro-physiological traits with significant correlations with the STI are shown in Figure 6. Generally, plants need to maintain positive shoot turgor to enable the growth of new tissues, a process that requires osmotic adjustment. Our results showed that the leaf sap osmolality was correlated negatively with salinity tolerance under both control and saline conditions (Table 3), which indicates that osmotic adjustment plays an important role in salt tolerance. Compared with the salt-sensitive genotypes, the salt-tolerant genotypes had a lower leaf osmotic pressure and a higher shoot water content (Figures 6B,E), indicating that the salt-tolerant genotypes had effective osmotic adjustment.

Furthermore, lower stomatal density was found in the salt-tolerant wheat than in the salt-sensitive genotypes (Figure 6C), and stomatal density had a significant positive correlation with leaf sap osmolality and a negative correlation with the shoot water content (all significant at *p <* 0.05; Figure 4). Taken together, these results suggest that salt-tolerant varieties maintain the water potential in the cells under high osmotic stress conditions through lower stomatal density (Munns et al., 2019, 2020). These findings confirm our previous observation of salt tolerance in other cultivars of wheat (Min Zhu and Sergey Shabala, unpublished results; Supplementary Table 3 and Figure 3), and this is also consistent with the findings for quinoa (Shabala et al., 2012, 2013) and barley (Kiani-Pouya et al., 2020). It has been reported that a freshwater species of *Spartina* had high stomatal density, while salt marsh species had significantly lower stomatal densities (Maricle et al., 2009). These observations emphasize the importance of reducing stomatal density as a stress-escaping strategy under saline conditions. It should be noted that the decrease in stomatal density is accompanied by a reduction in CO_2_ assimilation, ultimately resulting in lower yield or biomass (Hedrich and Shabala, 2018). Plants have evolved multifaceted adaptive strategies to cope with salinity damages; salt-tolerant varieties could utilize other mechanisms to compensate for yield and biomass losses. In this study, the higher shoot biomass accumulation of salt-tolerant varieties is sufficient to prove this (Figure 6F). Moreover, the higher leaf K^+^/Na^+^ discrepancy is the other distinction between tolerant and sensitive groups (Figure 6D), which could be one of the reasons why salt-tolerant genotypes maintain better growth even with reduced CO_2_ absorption. However, more evidence is required to clarify the underlying mechanism of this phenomenon.

A significantly negative correlation between salt tolerance and stomatal density was found in our study (significant at *p <* 0.05; Table 3), which is in contrast with previous research conducted on other cultivars of wheat (Zhu et al., 2014, 2016b). Salt tolerance is a complex trait that is related to a variety of biological processes, and significant genetic variability in salinity stress tolerance exists between genotypes. Therefore, different wheat varieties could display different strategies to cope with salt stress depending on the genetic background (ecotypes or genotypes) and the duration and intensity of the stress. The physiological basis for this genetic variability in the salinity tolerance in wheat, as well as in other species, is not fully understood.

### Leaf K^+^/Na^+^ as a Reliable Screening Index for Salt Tolerance, and the Na^+^ Exclusion Mechanism Affects Salt Tolerance in Wheat

In salt-stressed habitats, excessive Na^+^ concentration in the cytosol leads to the competition by Na^+^ for K^+^ binding sites, which can cause a degradation of chlorophyll and inhibit the normal functioning of a large number of enzymes and proteins (Isabelle et al., 2013; Anschütz et al., 2014). Therefore, the ability of plants to maintain an optimal K^+^/Na^+^ ratio is considered to be a key feature of salinity tolerance (Tester and Davenport, 2003; Ann et al., 2008). In our experiment with 30 wheat varieties, the relative leaf K^+^/Na^+^ ratio was the strongest determinant of salinity tolerance (Table 3). Tolerant genotypes were more efficient in maintaining higher leaf K^+^/Na^+^ ratios (Figure 6D), consistent with previous results for barley (Shabala et al., 2010). The steady-state of the K^+^/Na^+^ ratio can be achieved by restricting Na^+^ accumulation in the shoot or by improving K^+^ retention in the leaf mesophyll (Shabala et al., 2013; Zhu et al., 2016b); however, our data revealed no significant correlation between the leaf sap K^+^ content and salinity tolerance, whereas the leaf sap Na^+^ content was highly negatively correlated with the STI (Table 3). Hence, it can be concluded that a plant’s ability to exclude Na^+^ from the shoot, rather than delivering K^+^ to the shoot and retaining it in the mesophyll, makes a major contribution toward maintaining an optimal leaf K^+^/Na^+^ ratio and affecting the overall salinity tolerance of wheat. There were no correlations between the leaf Na^+^ concentration and salinity tolerance in the study of bread wheat (Genc et al., 2019), and it was noticeable that the loci for Na*^+^* exclusion resulted in just 18% variation in seedling biomass under salinity stress (Genc et al., 2016). This suggests that additional mechanisms such as tissue tolerance and osmotic adjustment need to be considered to breed wheat that is tolerant to salinity stress.

In the present investigation, salinity stress increased the relative expression of the *TaHKT1;5* and *TaSOS1* genes. The salt-tolerant variety showed greater increases in both gene relative expression compared with the salt-sensitive variety (Figure 7), which indicates that Na^+^ exclusion may be one of the key salinity tolerance mechanisms in wheat. The relative expression of *TaHKT1;5* showed a significant difference between the salt-tolerant genotype and the salt-sensitive genotype (significant at *p* < 0.05; Figure 7), suggesting that *TaHKT1;5* may play a more important role in Na^+^ exclusion compared with *TaSOS1* in plant shoots. Moreover, our results revealed that salinity stress increased the relative expression of *TaAKT1* (Figure 7), indicating that K^+^ retention may contribute to maintain the steady-state of the K^+^/Na^+^ ratio and enhance salinity tolerance in wheat. The relative expressions of *TaHKT1;5* and *TaAKT1* in the shoot of the salt-tolerant variety Huaimai 29 showed a significant difference in comparison to the salt-sensitive genotype Argine (significant at *p* < 0.05; Figure 7), indicating Na^+^ exclusion from the shoot, rather than the delivery of K^+^ to the shoot and retention in the mesophyll, resulting in a major contribution toward maintaining an optimal leaf K^+^/Na^+^ ratio, which confirms the results presented in Table 3.

## Conclusion

In this study, 18 parameters were used to evaluate the salt tolerance of 30 different wheat genotypes. Among them, stomatal density and the leaf K^+^/Na^+^ ratio significantly affected the salt tolerance index, which could be used as rapid and convenient screening indices for salinity stress tolerance in wheat at the seedling stage. Furthermore, our results suggested that salt-tolerant wheat had better Na^+^ exclusion and osmotic adjustment ability than the salt-sensitive wheat.

## Data Availability Statement

The original contributions presented in the study are included in the article/Supplementary Material, further inquiries can be directed to the corresponding author/s.

## Author Contributions

RT: investigation, formal analysis, and writing – original draft. JD, CL, and XZ: conceptualization and resources. MZ: editing, supervision, and project administration. WG: supervision and project administration. All authors have read and agreed to the published version of the manuscript.

## Conflict of Interest

The authors declare that the research was conducted in the absence of any commercial or financial relationships that could be construed as a potential conflict of interest.
