# Supplementary material for: Evaluating and Screening of Agro-Physiological Indices for Salinity Stress Tolerance in Wheat at the Seedling Stage
Source: Front Plant Sci. 2021 Mar 31;12:646175. doi: 10.3389/fpls.2021.646175 (PMC8044411; doi:10.3389/fpls.2021.646175)
Supplement: Supplementary file 1 [file Data_Sheet_1.pdf]

Suppl. Table 1. Preliminary screening trial in a glasshouse conducted in 2017-2018.

Genotypes	GR (C)/%	GR (S)/%	Chl	SC	Genotypes	GR (C)/%	GR (S)/%	Chl	SC
Argine	41	38	37	6	Ningmai 22	77	62	40	6
Buahin	98	98	22	7	Ningmai 23	82	64	39	4
DH16	82	84	16	5	Ningmai 24	86	88	48	7
Elnilein	97	96	41	7	Ruihua 520	63	53	42	5
G325	76	72	36	5	Shangnong 17	76	60	46	5
G406	84	76	33	6	Shangnong 20	92	84	48	7
G450	93	77	45	6	Shengxuan 6	63	49	48	7
Yangmai 11	74	61	48	7	Sumai 10	1	2	40	1
YF4	85	82	50	7	Sumai 11	2	6	-	-
Annong 0711	-	93	40	7	Sumai 188	-	100	47	7
Annong 1124	-	99	46	7	Sumai 9	84	74	48	7
Annong 1216	91	86	47	1	Wanmai 37	54	67	48	7
Baomai 2	96	90	41	7	Wannong 2	77	66	41	7
Baomai 5	78	52	45	5	Wannong 244	90	90	34	7
Baomai 6	62	43	48	7	Wannong 84	57	58	45	7
Emai 352	96	88	32	7	Weilai 0818	84	70	44	6
Emai 170	77	73	44	6	Womai 182	77	74	45	7
Emai 18	79	70	36	1	Womai 9	54	49	40	5
Emai 195	98	96	45	7	Xinong 979	61	48	42	7
Emai 23	86	70	43	2	Xianmai 8	93	93	44	5
Emai 251	83	73	30	7	Xiangmai 25	69	53	40	5
Emai 580	85	71	35	4	Xiangmai 35	95	90	37	7
Emai 596	88	77	43	4	Xiangmai D31	90	82	42	7
Fanmai 5	69	76	41	7	Xinmai 26	88	96	36	7
Fumai 1228	62	71	41	5	Xumai 33	88	89	39	7

(continued)

Genotypes	GR (C)/%	GR (S)/%	Chl	SC	Genotypes	GR (C)/%	GR (S)/%	Chl	SC
Gushen 6	92	89	42	3	Xumai 9158	99	89	39	7
Guanmai 1	90	73	49	5	Yannong 0999	96	92	47	7
Haomai 6	-	99	44	7	Yannong 19	86	86	34	5
Huacheng 3366	96	87	45	5	Yannong 999	96	92	47	7
Huamai 1168	73	63	47	7	YFM 1025	70	70	43	6
Huamai 368	87	86	43	7	YFM 2054	74	57	49	7
Huamai 6	70	87	32	7	YFM 4	-	97	50	7
Huamai 7	81	62	31	4	YFM 5	78	63	48	5
Huaimai 29	91	92	44	5	Yangmai 13	81	76	44	6
Huaimai 32	92	82	43	5	Yangmai 15	51	30	47	6
Huaimai 33	74	72	42	7	Yangmai 16	92	87	40	6
Huaimai 35	66	40	45	7	Yangmai 19	91	88	48	7
Jimai 22	45	21	46	7	Yangmai 20	74	58	37	6
Jiangmai 919	85	73	43	7	Yangmai 21	99	92	32	7
Lemai G1302	85	81	46	4	Yangmai 22	89	70	42	6
Lemai 608	96	84	44	6	Yangmai 23	84	92	50	6
Lianmai 7	87	86	47	6	Yangmai 25	92	93	43	7
Linagxing 99	87	79	45	6	Zhenmai 11	87	89	49	7
Longke 1221	35	32	46	5	Zhenmai 12	59	52	46	7
Luomai 10	79	79	40	5	Zhengmai 119	84	83	39	7
Mingmai 1	80	73	39	6	Zhengmai 9023	87	61	39	5
Ningmai 13	78	73	49	7	Zhongyu 1211	90	89	42	7
Ningmai 21	91	94	39	6	Zimai 19	73	53	45	6

GR (C), Germination rate under control; GR (S), Germination rate under under 150 mM NaCl stress; Chl, chlorophyll content (SPAD-values); SC, Survival score (0 = no symptoms of seedling damage; 10 = completely dead).

Suppl. Table 2. List of primers used in Real-Time qPCR.

Gene Name	Forward Primer	Reverse Primer	Amplicon Size (bp)
TaHKT1 ;5	ATGGGCCGGGTGAAAAGATT	TCCAGAAGGGGTGAACATGC	136
TaSOS1	TCGGACCATCACGAACACAG	CGTCATGCTGCCATACATGC	167
TaAKT1-like	GCCTAGCTGCGACGGATAAT	GGCAACGTCAAGAACCAACC	161
TaActin	AGGAGAAGCTCGCTTACGTG	GGGCACCTGAACCTTTCTGA	136

Suppl. Table 3. Genotypes ranking according to relative straw dry weight of 30 wheat varieties

	Genotypes	Relative straw dry weight		Genotypes	Relative straw dry weight		Genotypes	Relative straw dry weight
**S**	Covelle	0.28	**MT**	Jori	0.46	**T**	Kharchia 65	0.56
	Biskri ac2	0.34		Aus 12746	0.47		H7747	0.58
	Aus 16469	0.35		Cranbrook	0.47		13953	0.59
	Berkut	0.38		Krichauff	0.48		Opata	0.60
	India 38	0.42		Timilia	0.52		Janz	0.60
	Jandaroi	0.44		Citr 7792	0.52		Iran 118	0.68
	Kalka	0.45		Persia 21	0.52		Kukri	0.69
	Tamaori	0.45		Sokol	0.54		Gladius	0.78
	Iraq 43	0.45		Hyperno	0.54		Titmouse S	0.81
	Odin	0.45		Iraq 50	0.55		Zulu	0.81

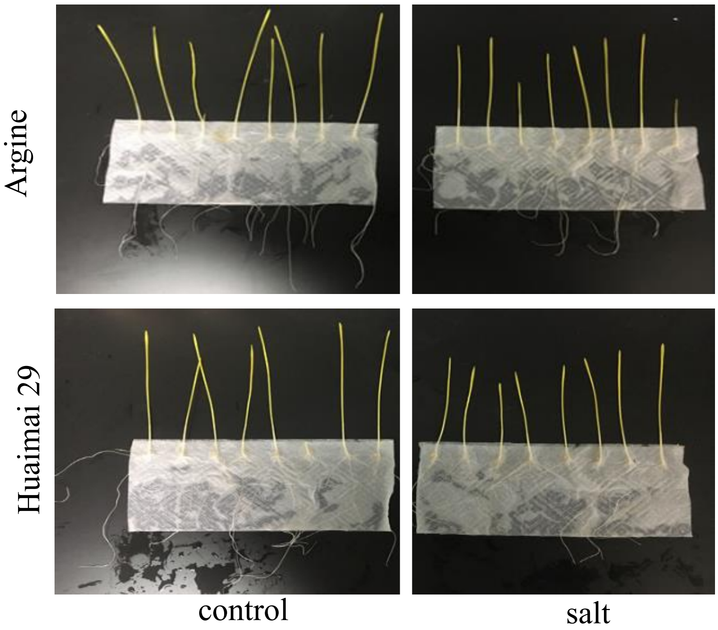

Suppl. Fig 1. Phenotypes of 7-d-old plants treated with 150mM NaCl for 24 h.

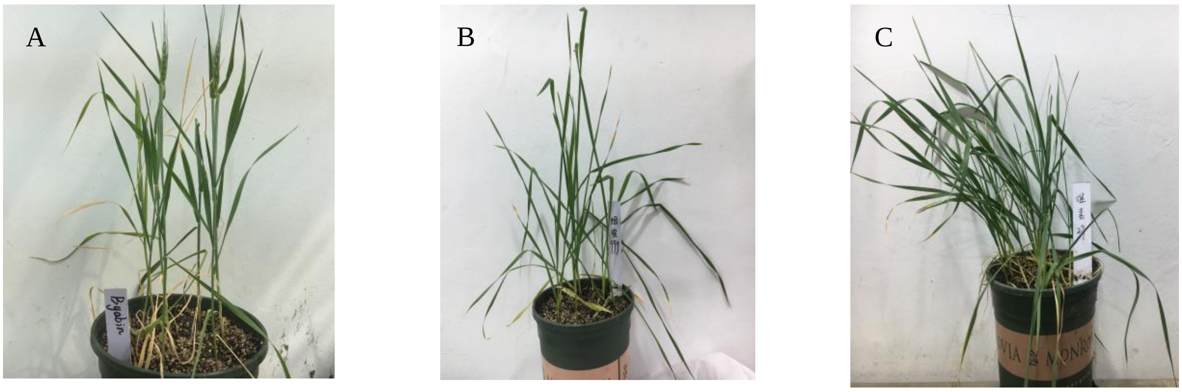

Suppl. Fig 2. Growth performances of different varieties under 150 mM NaCl stress. A: Buahin, Sensitive genotype (STI = 0.31); B: Yannong 999, moderately tolerant genotype (STI =0.51); C: Huaimai 29, tolerant genotype (T; STI =0.77).

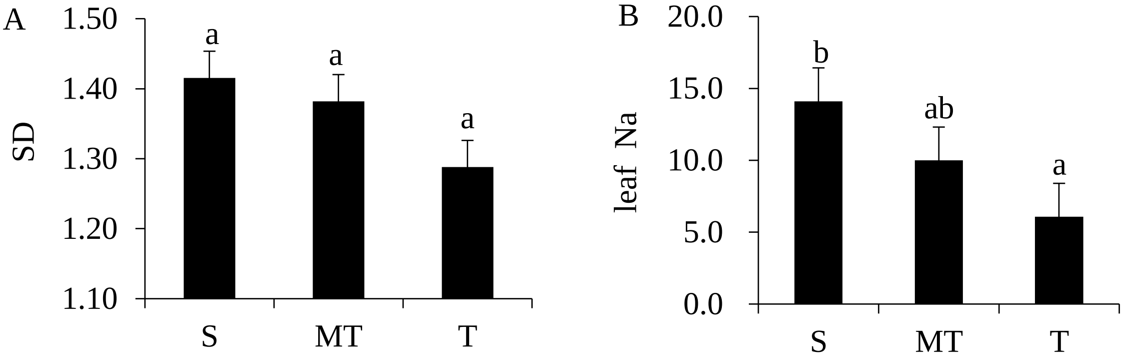

Suppl. Fig 3. Mean stomatal density (A) and leaf Na^+ content (B) for 30 wheat genotypes clustered according to their salinity stress tolerance. Cluster 1 = Sensitive (S; Relative straw dry weight < 0.45); Cluster 2 = moderately tolerant (MT; Relative straw dry weight 0.45-0.55); Cluster 3 = tolerant (T; Relative straw dry weight > 0.55) The scores are the relative values under saline treatment (% control) (R). Different lowercase letters indicate a significant difference ($P < 0.05$).
